# Factitious Hypoglycemia in an Infant With Undetectable Exogenous Insulin by a Commercial Insulin Immunoassay: A Diagnostic Pitfall

**DOI:** 10.7759/cureus.33224

**Published:** 2023-01-01

**Authors:** Hisham Arabi, Amir Babiker, Asma M Awadalla, Faroug Ababneh, Maha A AlMuneef

**Affiliations:** 1 Pediatrics, King Abdullah Specialist Children's Hospital, Riyadh, SAU; 2 Genetics and Precision Medicine, King Abdullah Specialist Children's Hospital, Riyadh, SAU

**Keywords:** analogue insulin cross-reactivity, insulin assay sensitivity, child protection, exogenous insulin, munchhausen by proxy, factitious hypoglycemia

## Abstract

Factitious hypoglycemia in infancy is a rare, life-threatening manifestation of Munchausen syndrome by proxy (MSBP). The hallmark of such presentation is the detection of low c-peptide combined with high insulin at the time of hypoglycemia. We report the case of a male infant who presented with recurrent severe unexplained hypoglycemic episodes since the age of six months. Two of his siblings had similar unexplained hypoglycemia episodes at a young age. He was extensively investigated, and all were normal, for endocrine and metabolic etiologies. He underwent fundoplication and insertion of a gastrostomy tube with multiple lengthy hospital admissions. His mother had diabetes and was on insulin treatment; she also had mental health issues with family-related social stressors. His hypoglycemic attacks resolved once separated briefly from his mother on the ward, raising our suspicion of MSBP. The exogenous administration of insulin was only confirmed following a scheduled change of our local Insulin assay in our laboratory when his insulin was detectable with low C-peptide on one of his typical attacks. Apparently, our previous insulin immunoassay lacked sensitivity for his mother’s long-acting insulin. We are reporting this case to raise awareness about this potential diagnostic pitfall.

## Introduction

Factitious hypoglycemia in infancy is a rare presentation of Munchausen syndrome by proxy (MSBP) [1-5]. The hallmark of such a diagnosis is a detectable disparity between C-peptide and insulin levels at the time of acute hypoglycemia [6]. Commercially available human insulin assays vary in their ability to detect commonly prescribed insulin analogs [7-11]. 

We are reporting a case of factious hypoglycemia in infancy caused by exogenous human insulin analog which was not detectable initially by our laboratory insulin assay. We aim to increase awareness about this diagnostic pitfall in such a scenario to avoid delayed diagnosis and unnecessary interventions.

## Case presentation

Our patient, a male infant, was born at 24 weeks gestation. His long neonatal course was complicated with grade II intraventricular hemorrhage, seizures, chronic lung disease, and necrotizing enterocolitis (conservatively managed). After five months in the neonatal intensive care unit (NICU), he was discharged on oral feeding, in room air, and on antiepileptic medication. Within the first few weeks after discharge, he had two admissions with seizures and apnea needing brief pediatric intensive care unit (PICU) admissions. No hypoglycemia was documented during those presentations.

**Figure 1:**
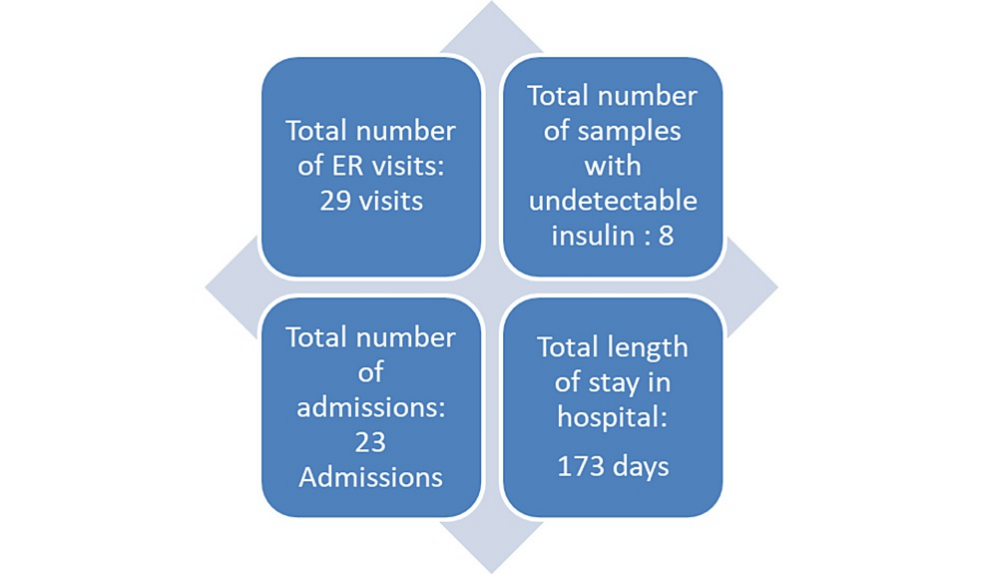
Hospital activities and length of stay related to hypoglycemia between ages six months to two years

This was followed by another admission, at the age of six months, to our general pediatric team with reported milk intolerance with vomiting and poor weight gain. He was treated for assumed gastroesophageal reflux disease (GERD) presentation with little improvement reported by his mother. On day 5 of his admission, his mother asked if we could check his glucose level as he was pale and sweaty. His previous random glucose readings were normal. The mother claimed that she has experience with hypoglycemia symptoms as two of his siblings had hypoglycemic episodes at a young age. His bedside glucose was low at 2.2 mmol/l. Since that event, recurrent severe hypoglycemia became his main medical problem leading to multiple ER presentations and lengthy hospital admissions (Figure:1). 

He was extensively investigated by the genetic/metabolic and endocrine teams. He was found to have non-ketotic hypoglycemia with undetectable insulin (insulin < 2.0 uIU/ml) on multiple critical samples with no other abnormalities. Radiological studies including upper gastrointestinal series (UGI) and milk scans showed features of gastroesophageal reflux. As a result of reported oral milk refusal at home with persistent vomiting and recurrent hypoglycemia episodes, he was kept on nasogastric tube feeding with GERD medical therapy for a few months. He eventually underwent insertion of a gastrostomy tube with Nissen’s fundoplication at the age of nine months. His feeding and blood glucose would typically stabilize while inpatient for a few days before his discharge, only to return a few days later with another hypoglycemia-related presentation (ranging from reported low blood glucose readings at home to active seizures). His hypoglycemia would typically be difficult to control in the first 24 hours despite 10% glucose infusion along with continuous feed (with added corn starch) reaching an estimated 12 mg/kg/minute of glucose load. His attacks had no specific pattern related to feeding interruption with limited evidence of intercurrent illnesses.

On two occasions, after a period of stabilization during admission, we attempted controlled fasting in the mother's presence. During the fasting test, he would only tolerate two hours of fasting before his blood glucose dropped.

The mother appeared to be caring and dedicated to his needs. She is a known patient of type 2 diabetes for 10 years with hypertension, both poorly controlled. She was on multiple medications including insulin detemir (Levemir®) as her long-acting insulin, as well as a beta-blocker and a calcium channel blocker. She disclosed to us that she suffers from depression and was on relevant medication on and off. Also, she told us that she is overwhelmed with a large family. According to the mother, the father tends to use violence to resolve familial issues and to discipline his children. She reported a history of domestic violence by the father towards her and the patient's older siblings, which was previously investigated by our Suspected Child Abuse and Neglect (SCAN) team. The patient's brother aged four years and sister aged 10 years both were reported to experience hypoglycemia episodes at a younger age. The four-year-old brother was investigated at our hospital before with no diagnosis for those attacks “now resolved”.

In view of the family history, a detailed metabolic and molecular workup was done, including whole exome sequencing followed by whole genome sequencing which all came back negative. With his recurrent non-ketotic hypoglycemia despite high glucose load with maternal access to insulin, the possibility of exogenous insulin was one of our differential diagnoses. However, all his critical samples showed undetectable insulin at the time of hypoglycemia. Our suspicions of this possibility increased after the child tolerated for the first time a brief fasting for four hours with stable glucose during a rare occasion without his mother’s presence.

The possibility of factitious hypoglycemia induced by the mother was considered by the primary team. Following a multidisciplinary review of his case led by the SCAN team, it was clear that all the involved specialties shared the same concern. Although we were lacking the evidence, with our high level of suspicion the SCAN team confronted the mother with our concerns.

We asked the mother to leave him in the hospital under the care of his older adult sister as a sitter. In the absence of his mother, the child was weaned from continuous feeding to bolus feeding and he tolerated prolonged fasting for 18 hours with no hypoglycemia. Input from the adult mental health team for the mother was arranged and a clear plan of close follow-up with the SCAN team, home health care team, and the family therapist was put in place before being discharged home under the care of his mother at that point.

Following that confrontation, the child had no attacks for two months. However, he presented again later with another hypoglycemia episode while under the care of his mother. A critical sample at the time of hypoglycemia revealed a low C-peptide level of 0.14 nmol/l (0.26-1.72 nmol/l) with high insulin level of 143 pmol/l (22.9-116.95 pmol/l) in keeping with exogenous insulin administration.

When we investigated this first high insulin reading further, we found that two changes took place before this presentation. The mother was reviewed by her diabetes team before his last discharge and her long-acting insulin was changed to glargine (Lantus®). Also, we came to know that our laboratory service had a scheduled change in their insulin immunoassay a month before this reading to a new assay, which is reported to have better cross-reactivity to synthetic insulin.

A confirmed diagnosis of factitious hypoglycemia induced by exogenous insulin was made. The mother was confronted with the new results. She never admitted to administering insulin; however, she did not challenge the allegations. She was the only caregiver with access to the child throughout his hospital admissions. On the balance of probability, we had sufficient proof to proceed with local child protection legal procedures led by the SCAN team to ensure the safety and wellbeing of our patient and his siblings. Over the following two years, the child had no further hypoglycemic attacks. He had only two brief hospital admissions with common childhood infections. He was gradually weaned from gastrostomy tube feeding to a normal oral diet. The family continues to be followed up by our SCAN team.

## Discussion

MSBP, also known as factitious disorder imposed on another (FDIA), is a relatively rare psychiatric disorder of the caregiver. It is a form of child abuse with a wide spectrum of presentations ranging from a mild form where the caregiver falsifies or exaggerates reported symptoms leading to unnecessary and potentially harmful medical interventions, to a severe end where the mother induces a potentially life-threatening illness using toxins or medications [12-14]. Induced hypoglycemia by exogenous insulin administration, as in our patient, is a severe form of MSBP with a high risk of mortality and morbidity [1-4].

Diagnosing MSBP syndrome can be challenging for most pediatricians as it requires a high level of suspicion. With complex presenting symptoms, a multidisciplinary approach would be desirable going through the stages of ruling out possible organic causes before focusing on the possibility of MSBP. Finding evidence to establish a solid diagnosis can be a difficult and lengthy process. The treating team is likely to go into a long phase of uncertainty, balancing the risk of losing the family's trust versus their obligation to keep the child safe [14-15]. 

Often exogenous insulin-induced hypoglycemia is easy to diagnose, with the typical finding of low C-peptide with detectable/high insulin at the time of hypoglycemia. In our case, the insulin was undetectable on multiple occasions.

Nevertheless, we had a strong clinical suspicion based on his recurrent non-ketotic hypoglycemia, and the need for a high glucose load to stabilize his glucose level during a typical attack with no other explanation after extensive metabolic and genetic workup. Furthermore, the maternal mental health issues, her social stressors, and her access to insulin with a previous unexplained similar presentation in his siblings support a possible diagnosis of MSBP.

Driven by our concern for the child’s safety, the mother was challenged with this possibility without solid evidence. A multidisciplinary approach led by the SCAN team at this point was very helpful to support the primary team to make such a challenge.

Commercially available insulin assays are frequently utilized to detect analog insulin in different clinical scenarios including our case. Although the limited ability of those assays in detecting commonly prescribed insulin analogs is well documented in the literature, it is still not common knowledge for physicians and even for laboratory personnel as it is not commonly provided by the manufacturers’ s operation instructions. Insulin analogs have a different primary sequence to human insulin, The more complex the sequence change in comparison to native human insulin, the less likely to have cross-reactivity with commercial human insulin assays [7-11].

In a study by Parfitt et al., they spiked pooled human serum samples with 15 different forms of exogenous insulin to test the detection rate of 10 major commercial insulin immunoassays in use in the UK. They reported high variability in the detection rate between those assays with four of them having no cross-reactivity to any synthetic insulin analog. Complex insulin, like insulin degludec (Tresiba®) and insulin detemir (Levemir®), was reported to have the least cross-reactivity across most assays. Our previously used local immunoassay was reported to have poor cross-reactivity to all synthetic insulin in this study [7].

In our case, we considered sending any future critical insulin sample to another laboratory. However, fortunately, it happened that our laboratory had a scheduled change to a new insulin assay, which is reported in Parfitt et al.'s study [7] to have moderate cross-reactivity to detemir (Levemir®) and good cross-reactivity to the mother’s newly introduced long-acting insulin glargine (Lantus®). With those changes in place, it was not surprising when, in his last presentation, the exogenous insulin was detectable confirming the diagnosis of MSBP.

## Conclusions

The possibility of exogenous administration of insulin should not be ruled out on the basis of undetectable insulin in the presence of a strong clinical suspicion of MSBP. Commercially available human insulin assays vary in their abilities to detect commonly prescribed insulin analogs. To our understanding, there is a lack of awareness among pediatricians about this diagnostic pitfall. Such an early consideration, in this life-threatening presentation of MSBP, could save the child lengthy unnecessary medical interventions and ultimately protect the child from ongoing abuse imposed on him by his caregiver's mental illness.
